# Partnerships Connecting Healthcare and Community-Based Organizations in Virginia

**DOI:** 10.1093/geroni/igaa057.1777

**Published:** 2020-12-16

**Authors:** Patricia Slattum, Pamela Parsons, Mary Rubino, Leland Waters

**Affiliations:** 1 Virginia Commonwealth University, Richmond, Virginia, United States; 2 Virginia Commonwealth, Richmond, Virginia, United States; 3 Family and Community Medicine, Eastern Virginia Medical School, Norfolk, Virginia, United States

## Abstract

The Virginia Geriatric Education Center (VGEC)’s Geriatrics Workforce Enhancement Program (GWEP) partners with two programs, Senior Strong at Eastern Virginia Medical School in Norfolk, VA and the Richmond Health and Wellness Program at Virginia Commonwealth University in Richmond VA to support their age-friendly initiatives. These programs enhance primary care for an older population experiencing adverse social determinants of health by providing screening around the 4Ms pillars of age-friendly healthcare and connecting participants with healthcare and community-based organizations. These programs offer a rich learning environment for interprofessional students. The VGEC GWEP strengthens these programs by developing faculty and student training in collaboration with the programs and facilitating program participation in the GWEP-CC Age-Friendly Action Community to develop and refine age-friendly practice workflows, referral pathways and documentation.

